# Two cases of intraoperative diagnosed Amyand hernia: Case report and literature review

**DOI:** 10.1016/j.ijscr.2024.109560

**Published:** 2024-03-22

**Authors:** Souad Ghattas, Jad El Bitar, Ribal aby Hadeer, Maher Salloum, Carl Akel, Henri Bitar

**Affiliations:** a Department of General Surgery - Mount Lebanon Hospital University Medical Center, University of Balamand, Beirut, Lebanon; b Faculty of Medicine and Medical Sciences, University of Balamand, El-Koura, Lebanon

**Keywords:** Amyand hernia, Right inguinal hernia, Mesh herniorrhaphy, Acute appendicitis

## Abstract

**Introduction:**

Amyand hernia is the presence of an incarcerated vermiform appendix (either inflamed or not) within the hernia sac. This type of hernia is very rare with an incidence reported to be 0.5 to 1 % and even rarer in adults.

**Cases presentation:**

We present here two cases of male patients found the have an Amyand Hernia diagnosed incidentally intraoperatively, and managed with appendectomy and mesh herniorrhaphy.

**Clinical discussion:**

For the management of this type of hernia, in general, the surgeon should perform an appendectomy with the repair to prevent future herniation or appendicitis, but some opinions differ, and state that when there are no signs of inflammation, it is not required to perform a preventative appendectomy.

**Conclusion:**

The decision on how to manage depends on multiple factors including inflammation of the appendix, the possibility of abdominal sepsis, and the patient comorbidities. The status of the appendix determines whether to undergo hernia repair with or without mesh.

## Highlights

• Amyand hernia is the presence of an incarcerated vermiform appendix within the hernia sac.
• This type of hernia is very rare with an incidence reported to be 0.5 to 1% and even rarer in adults.
• It’s difficult to diagnose preoperatively due to its uncomplicated presentation.
• The decision on how to manage depends on multiples factors including: inflammation of the appendix, the possibility of abdominal sepsis and the patient comorbidities.
• The status of the appendix determines whether to undergo hernia repair with or without mesh.

## 1 Introduction

Amyand hernia is the presence of an incarcerated vermiform appendix (either inflamed or not) within the hernia sac [1]. This type of hernia is very rare with an incidence reported to be 0.5 to 1 %. And is more common on in children around 3 times more than adults because of the patency of the processus vaginalis in children [2]. Losanoff proposed a classification that clarifies the advocated surgical treatment selections for different kinds of Amyand's hernia [1]. The work has been reported in line with the SCARE criteria [3].

We present here two cases of male patients found the have an Amyand Hernia diagnosed incidentally intraoperatively, and managed with appendectomy and mesh herniorrhaphy.

## 2 Case 1

A 64-year-old male patient with no known comorbidities and a past surgical history significant for open left inguinal mesh herniorrhaphy and prostatectomy presented to our surgical department with the chief complaint of swelling of the right inguinal region for the past 3 years increasing in size and discomfort lately. The patient had no signs or symptoms of bowel obstruction or strangulation.

On physical examination, vital signs were within normal limits. There was a bulge in the right inguinal region, above the right inguinal ligament, irreducible, fat-containing, smooth surface, normal skin, no local rise in temperature, non-tender, and soft in consistency. No obvious abnormalities were present in the left inguinal region. A computed enhanced tomography scan of the abdomen and pelvis was also done and confirmed the right inguinal fat-containing inguinal hernia.

The patient was scheduled for right open inguinal hernia mesh repair. Routine baseline laboratory investigations were within normal limits.

**Fig. 1:**
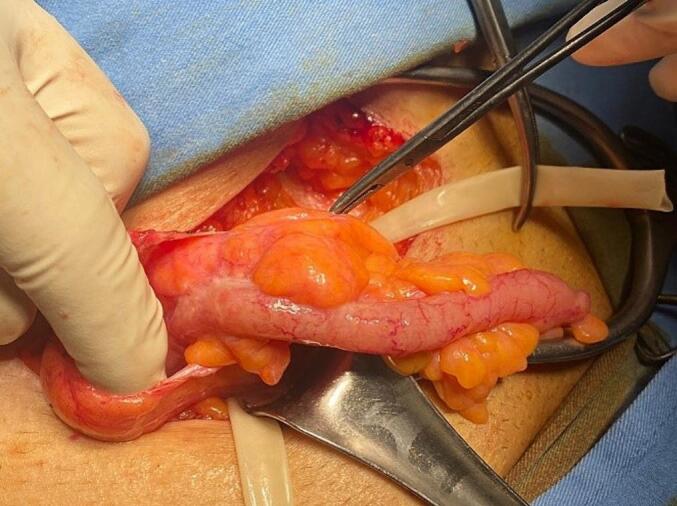
Appendix within the hernia sac.

During surgery, under spinal anesthesia, an indirect hernia sac was identified, after opening the hernia sac, the appendix was found protruding from the sac. The appendix was edematous and congested but not inflamed (Fig. 1). The decision was made to do an appendectomy inside the sac to avoid external spillage of fecal material. After ligation of the mesoappendix inside the sac, an appendectomy was performed. Then reduction of the sac content, lavage with betadine, high ligation division of the hernia sac, and Lichtenstein mesh hernioplasty were done.

Furthermore, histopathological examination of the appendix was suggestive of acute appendicitis without signs of malignancies.

The post-operative period was smooth and uneventful. The patient passed gas on day one post-operative, diet was started well tolerated and progressed. He was discharged home on day 2 post-operative on oral antibiotics and follow-up of the patient after 2 weeks remains unremarkable.

## 3 Case 2

A 60-year-old male patient with no known past medical and surgical history presented to our surgical department with the chief complaint of right scrotal swelling and right inguinal pain for the past 3 months. The patient had no signs and symptoms of bowel obstruction or strangulation.

On physical examination, there was an inguinal-scrotal bulge associated with testicular edema on the right side. On the left side, a small fat-containing inguinal hernia was palpated on the Valsalva maneuver. An Ultrasound was done and showed the presence of a right inguinal scrotal hernia sac, non-incarcerated or strangulated.

The patient was scheduled for bilateral open inguinal hernia mesh repair. Routine baseline laboratory investigations were within normal limits.

**Fig. 2:**
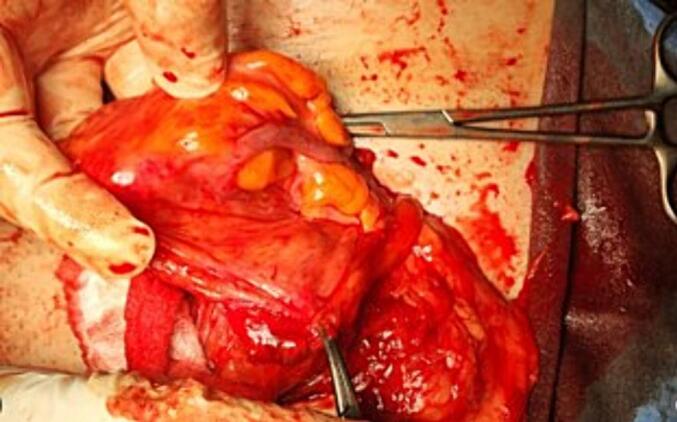
Appendix within the hernia sac and adherent to it.

During surgery, under spinal anesthesia, an indirect hernia sac was identified, after careful dissection to liberate the hernia sac and preserve the cord component, the hernia sac was opened, the appendix was found protruding from the sac and very adherent to it, separation couldn't be done (Fig. 2). The decision was made to do an appendectomy inside the sac to avoid external spillage of fecal material. After ligation of the mesoappendix inside the sac, an appendectomy was performed. Then reduction of the sac content, lavage with betadine, high ligation division of the hernia sac, and Lichtenstein mesh hernioplasty were done. Left Lichtenstein mesh hernioplasty was also, and a weak inguinal floor with a direct hernia was identified.

Furthermore, histopathological examination of the appendix was suggestive of congestive appendicitis with lymphoid follicle hyperplasia.

The post-operative period was smooth and uneventful. The patient passed gas on day one post-operative, diet was started well tolerated and the patient was discharged home with oral antibiotics, and follow-up of the patient after 2 weeks remains unremarkable.

## 4 Discussion

Amyand's hernia was first ever reported and performed by Claudius Amyand, who carried out the first appendectomy on a child who presented with a right non-reducible inguinal hernia containing his perforated appendix in 1735 [2]. This type of Hernia is very rare (incidence is around 0.5–1 %) but it has a preponderance in male children (it is mainly because of the patency of the processus vaginalis) compared to the other sex and other age groups, and in the right side where the appendix is usually found compared to the left [7]. However, in our cases, it was two male patients in the 7th decade of life and on the right side. Appendectomy was performed inside the sac—for the septic matter not spill out—along with the hernia repair since the appendix was edematous and to prevent future complications [5]. Nevertheless, it is the inflammatory state of the appendix that dictates the surgical strategy and type of hernia repair [8]. Losanoff and Basson have categorized 4 subtypes of Amyand's hernia depending on the status of the appendix in addition to their surgical approach (Table 1) [4]. Type I is a normal appendix: perform reduction or appendectomy with mesh hernioplasty. Type II is acute appendicitis localized in a hernial sac: perform appendectomy through the hernia, with mesh hernia repair; associated with a higher risk of mesh infection. Type III is acute appendicitis complicated by peritonitis: perform appendectomy through laparotomy; hernioplasty decision should be made based upon the spread of sepsis. Type IV is acute appendicitis accompanied by other abnormal pathology: hernioplasty may be contraindicated if damage is too extensive [4].

**Table 1:** Types of Amyand's hernia.

Types	Features	Surgical management
I	Normal appendix within an inguinal hernia	Depending on the patient's age, either a reduction or an appendectomy will be performed, followed by a hernioplasty using mesh.
II	Acute appendicitis localized within a hernia sac (no abdominal sepsis)	To treat a hernia, the surgeon will remove the appendix through the same incision, and repair the hernia without using any mesh.
III	Acute appendicitis within an inguinal hernia sac along with peritoneal sepsis	After removing the appendix via a large incision in the abdomen, the surgeon will repair the hernia directly without using mesh.
IV	Acute appendicitis within an inguinal hernia along with other related or unrelated abdominal pathology	The patient will undergo an appendectomy, and additional tests and treatments will be done based on their symptoms

For the management of this type of hernia, in general, the surgeon should perform an appendectomy with the repair to prevent future herniation or appendicitis, but some opinions differ, and state that when there are no signs of inflammation, it is not required to perform a preventative appendectomy [1]. The reason behind that was that an appendectomy increases the risk of infection to an otherwise sterile procedure [5]. However, whether an appendectomy is performed or not, the surgical procedure involves performing a hernia repair surgery, which may involve the use of prosthetic meshes. In case the appendix exhibits evident indications of chronic appendicitis, there is less disagreement regarding the appropriate treatment approach. In such instances, authors concur on the option of utilizing prosthetic materials for conducting the hernia repair surgery [4]. Our patients had an edematous and congested appendix without any features suggestive of acute appendicitis, and as a result, they were treated as a case of Type 1 Amyand hernia (as per the classification of Losanoff and Basson) and underwent an appendectomy with mesh repair. However, concerning the type 1 Amyand hernia, the decision to perform an appendectomy or retain the normal appendix has not yet been established [6].

Desai et al. state that the traditional practice discourages the use of mesh in cases where the appendix is inflamed or perforated, and instead, primary tissue repair is recommended [2]. Nevertheless, Kose et al. affirm that many researchers have reported the utilization of mesh in cases of appendectomy for inflamed appendices [6]. Notably, there have been no reported complications associated with the use of mesh in the surgical treatment of Amyand hernia. Thus, mesh inguinal hernia repair can be considered a safe technique when combined with appendectomy, regardless of whether the appendix is inflamed or not.

Shen Hang Han et al. proposed a total laparoscopic treatment strategy for Amyand's hernia. However, a prerequisite was that a preoperative diagnosis must be made. [9]. The advantages of laparoscopic surgery in such hernias are that there are low recurrence rates and it is associated with substantially less pain in the immediate postoperative period with earlier return to normal activities compared to open repair. A minimally invasive technique in inflamed hernias allows for an easier approach to the base of the appendix for firing the stapler. In situations when it was not feasible to accomplish the surgery completely laparoscopically, it was possible to mobilize the contents, and thereby reduce the extent of abdominal incision needed to complete the surgery [10].

## 5 Conclusion

Amyand's hernia is a rare presentation of inguinal hernias and is difficult to diagnose preoperatively due to its uncomplicated presentation. The decision on how to manage depends on multiple factors including inflammation of the appendix, the possibility of abdominal sepsis, and the patient comorbidities. The status of the appendix determines whether to undergo hernia repair with or without mesh.

## CRediT authorship contribution statement

Souad Ghattas (First Author), Jad El Bitar (Co-Author), Ribal aby Hadeer (Co-Author), Maher Salloum (Co-Author), Carl Akel (Co-Author), Henri Bitar (Corresponding Author).

## Declaration of competing interest

The authors report no conflict of interest.

## Data Availability

All data available upon request at Department of General Surgery, Mount Lebanon Hospital University Medical Center, University of Balamand, Beirut, Lebanon where the work was done.
